# Effects of ozone combined with articular injection of sodium hyaluronate on patients with knee osteoarthritis and their inflammatory factors and hemorheological indices

**DOI:** 10.4314/ahs.v23i2.40

**Published:** 2023-06

**Authors:** Chengyong Yang, Qun Chen

**Affiliations:** Department of Orthopedics, Affiliated Hospital of Yangzhou University, Yangzhou 225000, Jiangsu Province, China

**Keywords:** Ozone, sodium hyaluronate, knee osteoarthritis, inflammatory factor, hemorheological index

## Abstract

**Objective:**

Knee osteoarthritis (KOA) is a common chronic progressive disease. We aimed to evaluate the effects of ozone combined with articular injection of sodium hyaluronate on KOA patients and their inflammatory factors and hemorheological indices.

**Methods:**

A total of 292 KOA patients treated from October 2020 to October 2021 were randomly divided into observation and control groups (n=146). Observation group was treated with ozone combined with articular injection of sodium hyaluronate, while control group was routinely given glucosamine hydrochloride tablets and articular injection of sodium hyaluronate. The treatment was performed once a week for 5 consecutive weeks. Their general data, treatment outcomes, visual analogue scale (VAS) score, Hospital for Special Surgery (HSS) knee score, inflammatory factor levels and hemorheological indices were compared.

**Results:**

After treatment, observation group had significantly lower VAS score and higher HSS score than those of control group (P<0.05). The total response rate of observation group was higher than that of control group (P<0.05). The levels of inflammatory factors in the joint fluid were significantly lower in observation group than those in control group (P<0.05). The hemorheological indices were improved in both groups, especially in observation group (P<0.05).

**Conclusion:**

Ozone combined with articular injection of sodium hyaluronate has obvious therapeutic effects on KOA.

## Introduction

Knee osteoarthritis (KOA) is a common chronic progressive disease, with the pathological basis of degenerative lesions in knee cartilage or secondary hyperostosis^1^-^3^. The key in the treatment of KOA is to prevent and control the progression of disease, restore the knee joint function, and relieve the pain. Drug therapy, non-drug therapy and surgery are three commonly used clinical treatment methods currently, and articular injection of drug is the most important method^4^. In recent years, ozone interventional therapy has been employed to effectively alleviate pain caused by acute soft tissue injury. Moreover, it has obvious therapeutic effects on chronic soft tissue injury, with low treatment expenses^5^. Until now, the therapeutic effects of ozone combined with articular injection of sodium hyaluronate on inflammatory factors and hemorheology in the case of KOA remain largely unknown. In this study, therefore, the clinical efficacy of ozone combined with articular injection of sodium hyaluronate on KOA and its effects on inflammatory factors and hemorheology in patients were explored, aiming to provide references for the clinical treatment of KOA.

## Materials and Methods

### General data

A total of 292 KOA patients treated from October 2020 to October 2021 were selected and divided into observation and control groups (n=146) using a random number table. In observation group, there were 38 males and 108 females aged 61-81 years old, with an average of (67.22±8.31) years. The course of disease was 1-10 years, with an average of (5.43±2.12) years. In control group, there were 44 males and 102 females aged 65-85 years old, with an average of (66.99±8.41) years. The course of disease was 1-11 years, with an average of (5.51±2.21) years. Gender, age, weight, course of disease and lesion site had no significant differences between the two groups (P>0.05) (Table 1).

**Table 1:** General data

Data	Observation group (n=146)	Control group (n=146)	P
Gender			>0.05
Male	38	44	
Female	108	102	
Age (Y)	67.22±8.31	66.99±8.41	>0.05
Weight (kg)	63.82±4.69	64.15±4.76	>0.05
Course of disease (Y)	5.43±2.12	5.51±2.21	>0.05
Lesion site			>0.05
Left	80	76	
Right	66	70	

The diagnostic criteria for KOA developed by the American College of Rheumatology were used: 1) patients aged above 40 years old; 2) those with knee pain symptoms most of the time within 1 month; 3) those with morning stiffness for less than 30 min; 4) those with friction sound in the joints during movement; 5) those with symptoms of osteoarthritis in the synovial fluid examination; 6) those with osteophytes on the joint edges shown in X-ray. The cases simultaneously meeting the above criteria 1) 2) 3) 4) or 2) 6) or 2) 3) 4) 5) can be diagnosed as KOA.

**Inclusion criteria: 1)** patients meeting the above diagnostic criteria and with disease in one knee; 2) those who and whose families voluntarily participated in this study and signed for confirmation.

**Exclusion criteria: 1)** patients who used corticosteroids or non-steroidal anti-inflammatory drugs, immunopotentiator, immunosuppressor, immunomodulator, other decoctions or Chinese patent medicines, or underwent physical therapy within the last month; 2) those with obvious narrowing of diseased joint space, interarticular bone bridge formation, bone articular calcification or hollow state of joint space shown in CT examination; 3) those with rheumatism, visceral disease or tumours; 4) those complicated with congenital deformities of limbs, skin diseases or acute local trauma in knee joints; 5) those with mental illness or dysfunction of consciousness.

This study has been reviewed and approved by the Medical Ethics Committee of our hospital. Written informed consents were obtained from all patients.

### Treatment methods

Under a supine position, the knee joint was exposed and kept flexed at 70-90°, and the medial-lateral knee eyes were used as the puncture point. After routine disinfection and draping, the patients received infiltration anesthesia with 1% lidocaine injected layer by layer at the puncture point. The 7# puncture needle was inserted into the knee articular cavity from the puncture point, the needle cylinder was connected to draw out joint effusion, and the needle was retained, followed by articular injection of drugs. In control group, routine comprehensive therapy was performed: Glucosamine Hydrochloride Tablets (Zhejiang Chengyi Pharmaceutical Co., Ltd., NMPN H20060748) were taken once a day (1.5 g/time), and sodium hyaluronate (Shandong Bausch & Lomb Freda Pharmaceutical Co., Ltd., NMPN H10960136) was injected into the articular cavity (20 mg/time). In observation group, 30 mL of medical ozone at a concentration of 30 µg/mL was first injected into the knee articular cavity, and then the puncture mouth was covered with aseptic dressing for half an hour. Then 20 mg of sodium hyaluronate was injected into the knee articular cavity. The treatment was performed once a week for 5 consecutive weeks as one course in both groups. Patients were asked to avoid activities that damage the knee joints during treatment, such as mountain climbing and stair climbing.

### Observation indices

The degree of pain was assessed using the visual analogue scale (VAS) score before and after treatment, and the knee joint function was evaluated using the Hospital for Special Surgery (HSS) knee score. The overall therapeutic effect was evaluated according to the therapeutic effect criteria for KOA developed by the Society of Acu-potomology, China Association of Chinese Medicine in 2008. Before and after treatment, 3-5 mL of joint fluid was drawn from each patient, and the levels of vascular endothelial growth factor (VEGF), tumor necrosis factor-α (TNF-α), interleukin-1β (IL-1β), IL-6, high-sensitivity C-reactive protein (hs-CRP) and matrix metalloproteinase-13 (MMP-13) were detected via enzyme-linked immunosorbent assay (ELISA). Besides, hemorheological indices (erythrocyte sedimentation rate, hematocrit, erythrocyte aggregation index, fibrinogen, plasma viscosity, low-shear whole blood viscosity and high-shear whole blood viscosity) were measured with a hemorheology instrument.

### Evaluation criteria

In terms of VAS score, 0 point: no pain; 1-3 points: mild pain; 4-6 points: moderate pain; 10 points: severe pain. A higher score means severer pain. For HSS score, the total scores of pain, mobility, function, joint stability, flexion deformity and muscle strength are 30, 18, 22, 10, 10 and 10 points, respectively, and the full score is 100 points. A higher score represents better knee joint function. The efficacy was evaluated as follows: clinical control: The clinical symptoms such as pain and swelling disappeared, the knee joint motion returned to normal, the patient could stand and walk independently, and there was no recurrence after drug withdrawal; markedly effective: The clinical symptoms such as pain and swelling were improved significantly, the knee joint function was basically restored without affecting daily work and life; effective: Both pain and knee joint function were improved; ineffective: The clinical symptoms such as pain and swelling had no improvement, and the joint motion was restricted or even became worse. Total response rate - (clinical control + markedly effective + effective)/total cases × 100%.

### Statistical analysis

SPSS18.0 software (IBM Inc., USA) was used for statistical analysis. Measurement data were expressed as -(χ ± s). Independent t test was used for intergroup comparison, while paired t test was conducted for comparison at different time points. Numerical data were expressed as rate (%), and χ^2^ test was performed for intergroup comparison. P<0.05 was considered statistically significant.

## Results

### VAS and HSS scores

Before treatment, VAS score and HSS score had no significant differences between the two groups (P>0.05). Observation group had a significantly lower VAS score and a higher HSS score than control group after treatment (P<0.05). Besides, the VAS score declined and HSS score rose in both groups after treatment compared with those before treatment (P<0.05) (Table 2).

**Table 2:** VAS and HSS scores

Item	Observation group (n=146)	Control group (n=146)	P
VAS (point)			
Before treatment	7.64±1.11	7.59±1.12	>0.05
After treatment	2.84±0.51*^#^	4.16±0.91*	<0.05
HSS (point)			
Before treatment	58.19±7.61	57.78±7.51	>0.05
After treatment	83.61±9.21*^#^	71.12±8.44*	<0.05

* P<0.05 *vs*. before treatment

# P<0.05 *vs*. control group

### Treatment outcomes

The total response rate after treatment in observation group (97.26%) was significantly higher than that in control group (76.71%) (P<0.05) (Table 3).

**Table 3:** Treatment outcomes

Item	Observation group (n=146)	Control group (n=146)	P
Clinical control (n)	112	84	
Markedly effective (n)	26	22	
Effective (n)	4	6	
Ineffective (n)	4	34	
Total response rate (%)	97.26	76.71	<0.05

### Levels of inflammatory factors

Before treatment, no significant differences were found in the levels of inflammatory factors VEGF, TNF-α, IL-1β, IL-6, hs-CRP and MMP-13 in the joint fluid between the two groups (P>0.05). After treatment, the levels of the above inflammatory factors were significantly lower in observation group than those in control group (P<0.05), and they declined in both groups compared with those before treatment (P<0.05) (Table 4).

**Table 4:** Levels of inflammatory factors

Item	Observation group (n=146)	Control group (n=146)	P
VEGF (µg/L)			
Before treatment	26.81±3.12	27.13±3.22	>0.05
After treatment	15.22±1.75*^#^	25.23±2.81*	<0.05
TNF-α (pg/mL)			
Before treatment	57.07±11.93	56.82±11.71	>0.05
After treatment	25.45±6.71*^#^	39.66±8.92*	<0.05
IL-1β (pg/mL)			
Before treatment	35.12±6.42	34.93±6.32	>0.05
After treatment	17.23±3.02*^#^	27.35±4.71*	<0.05
IL-6 (pg/mL)			
Before treatment	111.57±26.01	109.72±25.85	>0.05
After treatment	54.29±12.31*^#^	82.46±18.92*	<0.05
hs-CRP (mg/L)			
Before treatment	9.12±1.22	9.15±1.15	>0.05
After treatment	4.75±0.81*^#^	6.81±0.92*	<0.05
MMP-13 (ng/mL)			
Before treatment	143.72±20.51	145.25±21.32	>0.05
After treatment	87.85±14.64*^#^	115.74±18.91*	<0.05

* P<0.05 *vs*. before treatment

# P<0.05 *vs*. control group

### Hemorheological indices

The hemorheological indices (erythrocyte sedimentation rate, hematocrit, erythrocyte aggregation index, fibrinogen, plasma viscosity, low-shear whole blood viscosity and high-shear whole blood viscosity) had no significant differences between the two groups before treatment (P>0.05). After treatment, the above indices were significantly lower in observation group than those in control group (P<0.05), while they declined in both groups compared with those before treatment (P<0.05) (Table 5).

**Table 5:** Hemorheological indices

Item	Observation group (n=146)	Control group (n=146)	P
Erythrocyte sedimentation rate (mm/h)			
Before treatment	9.91±1.62	9.96±1.54	>0.05
After treatment	7.24±0.81*^#^	8.82±1.14*	<0.05
Hematocrit (%)			
Before treatment	41.94±3.85	42.02±4.05	>0.05
After treatment	37.82±3.21*^#^	40.13±3.72*	<0.05
Erythrocyte aggregation index			
Before treatment	5.74±0.75	5.65±0.76	>0.05
After treatment	4.39±0.42*^#^	5.08±0.65*	<0.05
Fibrinogen (g/L)			
Before treatment	4.35±0.41	4.22±0.44	>0.05
After treatment	2.72±0.21*^#^	3.65±0.32*	<0.05
Plasma viscosity (ms/s)			
Before treatment	2.26±0.06	2.32±0.08	>0.05
After treatment	1.63±0.07*^#^	1.84±0.07*	<0.05
Low-shear whole blood viscosity (ms/s)			
Before treatment	11.72±1.32	11.51±1.34	>0.05
After treatment	9.64±1.05*^#^	10.43±1.25*	<0.05
High-shear whole blood viscosity (ms/s)			
Before treatment	6.12±0.51	6.03±0.52	>0.05
After treatment	5.04±0.41*^#^	5.64±0.53*	<0.05

* P<0.05 *vs*. before treatment

# P<0.05 *vs*. control group

## Discussion

KOA, also known as degenerative, hyperplastic or hypertrophic arthritis, is common among middle-aged and elderly women6. With further development, joint deformity and dysfunction occur in severe cases^7^. Currently, the pathogenesis of KOA remains unclear yet, but some scholars argue that long-term overload wear of articular cartilage, decreased secretion of synovial fluid nourishing joints, estrogen and endocrine dysfunction are all important pathogenic factors in middle-aged and elderly people^8^. At present, the primary goal of clinical treatment of KOA is to relieve pain, correct deformity and improve function, so conservative medical treatment is commonly used^9^. Endogenous sodium hyaluronate exert nutritional protective effects on joints. Due to joint synovial B cell apoptosis in patients with KOA, insufficient synthesis or degradation of sodium hyaluronate in the synovial fluid is caused, so the articular cartilage loses its protection, leading to increasing degeneration. After corrosion damage. Therefore, both pain and dysfunction occur in patients^3^. Ozone can resist the synthesis and secretion of pain-causing inflammatory factors, thereby easing pain. It can be decomposed into oxygen at room temperature to raise the local oxygen partial pressure in the body. In addition, it enhances the free-radical scavenging effect by increasing the content of superoxide dismutase so as to alleviate the damage of articular cartilage, ultimately exerting an anti-inflammatory effect. It also promotes the regeneration and repair of articular cartilage in the articular cavity after environmental changes, thus delaying the degeneration of knee joint^10^,^11^.

In the present study, there were no significant differences in general data between the two groups (P>0.05). Before treatment, VAS score and HSS score had no significant differences between the two groups (P>0.05). After treatment, the VAS score significantly declined and the HSS score significantly rose in both groups (P<0.05). It can be seen that both intra-articular injection of sodium hyaluronate and ozone therapy can greatly reduce the pain and ameliorate the knee joint function of KOA patients. Observation group had a significantly lower VAS score and a significantly higher HSS score than control group after treatment (P<0.05), suggesting that ozone combined with intra-articular injection of sodium hyaluronate has a better effect than injection of sodium hyaluronate alone. The total response rate after treatment in observation group was higher than that in control group (P<0.05), further confirming that ozone combined with sodium hyaluronate has a more significant effect. It has been proved that the inflammatory response is the main pathological feature of KOA, and inflammatory factors are the main factors destroying articular cartilage and inducing pain, whose levels, therefore, play an important role in the progression of disease. VEGF is able to increase the permeability of micro vessels and venules, and promote the formation of synovial inflammatory tissues and angiogenesis, thereby inducing inflammation and causing joint deformity^12^. TNF-α can increase vascular permeability through stimulating cartilage degrading enzymes, so that the local inflammatory response and edema are aggravated in the knee joint^13^. Moreover, IL-1β stimulates the synovium and chondrocytes to raise the expression of MMP, and then MMP results in the enhanced activity of protein-lysing enzymes in the cartilage matrix, and the cleavage of collagen network. As a member of the MMP family, MMP-13 possesses a potent cleavage effect on type II collagen, and it has been confirmed to be closely related to the destruction of articular cartilage^14^. Besides, IL-6 mainly secreted by the synovial lining cells in osteoarthritis inhibits the synthesis of proteoglycans in articular chondrocytes, and accelerates the degradation of cartilage matrix, ultimately worsening articular cartilage damage^15^. Hs-CRP secreted by hepatocytes is a non-specific inflammatory mediator, which exacerbates the articular cartilage degeneration through destroying the articular cartilage matrix^16^. In this study, no significant differences were found in the levels of inflammatory factors in the joint fluid before treatment between the two groups (P>0.05). After treatment, the levels of the inflammatory factors in the joint fluid were significantly lower in observation group than those in control group (P<0.05), and they all declined in both groups compared with those before treatment (P<0.05). It can be inferred that ozone and sodium hyaluronate can lower the levels of VEGF, TNF-α, IL-1β, IL-6, hs-CRP and MMP-13, thereby relieving the inflammatory response. The reason is that sodium hyaluronate may, through inhibiting the accumulation and reducing the concentration of inflammatory factors, alleviate the inflammatory injury, and improve the physicochemical state of synovial fluid in the articular cavity, thereby promoting articular cartilage repair. Therefore, injection of sodium hyaluronate alone can also reduce the pain and ameliorate the knee joint function of patients. Ozone has anti-inflammatory and analgesic effects, and it can promote the articular cartilage regeneration and repair. Therefore, ozone combined with intra-articular injection of sodium hyaluronate is more effective in reducing the levels of inflammatory factors, mitigating the pain, inhibiting the progression of disease and improving the joint function.

Osteoarthritis patients have increased blood viscosity and weakened erythrocyte deformability, and such a blood stasis state may enhance the progression of disease^17^. It is believed in modern medicine that the main primary factor of KOA is that the circulatory resistance around the joints is increased by both intraosseous hypertension and knee venous stasis, so that articular cartilage degeneration becomes worse, synovial edema and hyperostosis are caused, and lesions occur in bone and cartilage due to lack of nutrition^18^. In the present study, the hemorheological indices had no significant differences between the two groups before treatment (P>0.05), while they were all improved in both groups after treatment, more significantly in observation group (P<0.05). It can be inferred that sodium hyaluronate and ozone can inhibit the occurrence and development of KOA through improving hemorheological indices in varying degrees, and the combination of the two has a better effect.

In conclusion, ozone combined with intra-articular injection of sodium hyaluronate has a more significant therapeutic effect on KOA than injection of sodium hyaluronate alone, which can effectively lower the levels of inflammatory factors (VEGF, TNF-α, IL-1β, IL-6, hs-CRP and MMP-13), alleviate the inflammatory response and improve hemorheology, thereby reducing the pain and improving the knee joint function of patients. Regardless, this study is still limited. This is a single-centre study with a small sample size, so the results may have bias. Multicentre studies with larger sample sizes are ongoing in our group.
